# Combined use of insulin eye drops and cross-linked hyaluronic acid canalicular gel for the management of severe neurotrophic keratopathy

**DOI:** 10.1016/j.ajoc.2026.102637

**Published:** 2026-07-29

**Authors:** Filippo Lixi, Camilla Bianchi, Claudia Corda, Mario Verdiglione, Giulia Coco, Giuseppe Giannaccare

**Affiliations:** a Eye Clinic, Department of Surgical Sciences, University of Cagliari, Cagliari, Italy; b Department of Medical Sciences and Public Health, University of Cagliari, Monserrato, Italy; c Compounding Pharmacy, Farmacia Europea, Catanzaro, Italy; d Ophthalmology Unit, Department of Clinical Sciences and Translational Medicine, University of Rome Tor Vergata, Rome, Italy

**Keywords:** Neurotrophic keratopathy, Persistent epithelial defect, Topical insulin, Lacrifill, Canalicular gel

## Abstract

**Purpose:**

To describe the outcome of a novel combined therapeutic approach encompassing topical insulin eye drops and cross-linked hyaluronic acid canalicular gel in a case of severe recalcitrant neurotrophic keratopathy (NK) complicated by keratolysis.

**Case presentation:**

A 52-year-old male presented with a recalcitrant Mackie stage III post-herpetic NK in the right eye, characterized by a large persistent epithelial defect (PED) (area of 49.7 mm^2^), central stromal melting, corneal anesthesia, and neovascularization. Best-corrected visual acuity (BCVA) was reduced to hand motion. The new therapeutical approach included the withdrawal of previous therapies and the initiation of topical insulin (1 unit/mL, 4 times daily) combined with the insertion in the lower punctum of cross-linked hyaluronic acid canalicular gel (Lacrifill, Nordic Pharma, Inc., Berwyn, PA, US) to enhance ocular surface drug retention. In the subsequent follow-up visits, the PED area progressively decreased to 19.6 mm^2^ at 2 weeks and 4.4 mm^2^ at 4 weeks; complete re-epithelialization was achieved at 8 weeks. In parallel, BCVA improved to counting fingers at 1 m. No adverse events were reported.

**Conclusions:**

The combined use of topical insulin and cross-linked hyaluronic acid canalicular gel allowed achievement of complete corneal healing in a case of severe recalcitrant NK complicated by keratolysis. The regenerative properties of insulin eye drops were enhanced by the concomitant insertion of the occlusive device. This approach represents a rational, accessible, and well-tolerated therapeutic strategy that warrants further investigation in prospective controlled studies.

## 1 Background

Corneal epithelium, the outermost layer of the cornea, plays a crucial role in protecting against environmental insults and maintaining a smooth refractive surface, contributing to both ocular defense and optical clarity.^1^ This protective role is carried out through the function of corneal nerves, which are crucial for the regulation of cellular proliferation. Several conditions may impair the physiological corneal environment, causing epithelial disruption.^1^^,^^2^ Among these, neurotrophic keratopathy (NK) represents a corneal disease caused by loss of neurotrophic support that results in decreased or absent corneal sensation and several possible sequelae.^3^ Indeed, NK usually begins with pathological epithelial changes, including erosions and persistent epithelial defects (PEDs), defined as failure of complete corneal re-epithelialization within approximately 10-14 days despite appropriate conventional therapy.^4^ When the underlying corneal stroma becomes involved, opacification, stromal melting (keratolysis), thinning, or even perforation may occur.^3^ In cases of NK, early diagnosis and treatment are essential to promote epithelial healing, preventing complications, and, where feasible, restore corneal nerve function.^5^ Medical therapy is generally based on a stepwise approach involving discontinuation of toxic drugs, preservative-free tear substitutes, bandage contact lens, punctum occlusion; in more severe cases, blood- or amniotic-derived topical products and recombinant human nerve growth factor (rhNGF) eye drops can be necessary. When medical therapy fails, surgical procedures such as tarsorrhaphy, amniotic membrane transplantation, keratoplasty and corneal neurotization represent the last resort treatments.^5^^,^^6^

In recent years, insulin eye drops have increasingly emerged as a valuable therapeutic option in the management of moderate-to-severe NK, demonstrating promising outcomes.^7^, ^8^, ^9^ Although the precise mechanisms underlying its efficacy have yet to be fully elucidated, current evidence suggests that the beneficial effects may be attributable to the promotion of corneal nerve regeneration and/or the enhancement of epithelial cell migration.^10^ A novel canalicular gel occlusive device made of cross-linked hyaluronic acid (HA) has been recently designed to block tear drainage by occluding the canalicular system and to improve tears retention over the ocular surface.^11^ The gel-like material completely fills the lumen, preventing tear flow through the lacrimal drainage system representing a comfortable, durable, and biocompatible alternative to conventional silicone plugs.^12^^,^^13^

Herein, the successful outcomes of a severe recalcitrant NK treated with a 2-month course of insulin eye drops combined with the insertion of cross-linked HA canalicular gel are described.

## 2 Material and methods

Insulin eye drops (1 unit per mL) were prepared by a compounding pharmacy (Farmacia Europea, Catanzaro, Italy) in a grade D cleanroom, as previously described.^14^ A cross-linked HA canalicular gel occlusive device (Lacrifill, Nordic Pharma, Inc., Berwyn, PA, US) was inserted according to the manufacturer's guidelines.^11^ Briefly, following the instillation of topical anesthesia, the lower punctum was irrigated with saline solution to verify canalicular patency. Subsequently, a cannula connected to a pre-filled syringe containing the cross-linked HA filler was carefully introduced into the lower punctum, and a volume of 0.2 mL of cross-linked HA filler was injected into the canaliculus. At baseline and at each follow-up visit (biweekly), the following parameters were measured: i) area of the PED (mm^2^) from slit-lamp photographs taken with blue/yellow filters using image analysis with ImageJ software 1.53t (National Institutes of Health, Bethesda, MD; available at http://imagej.nih.gov/ij); ii) corneal sensitivity (mm) using the Cochet-Bonnet esthesiometer; iii) corneal thinnest point (CTP) (μm) using the anterior segment-optical coherence tomography (AS-OCT); iv) conjunctival hyperemia (Efron grade); v) best-corrected visual acuity (BCVA).

## 3 Case Presentation

**Fig. 1:**
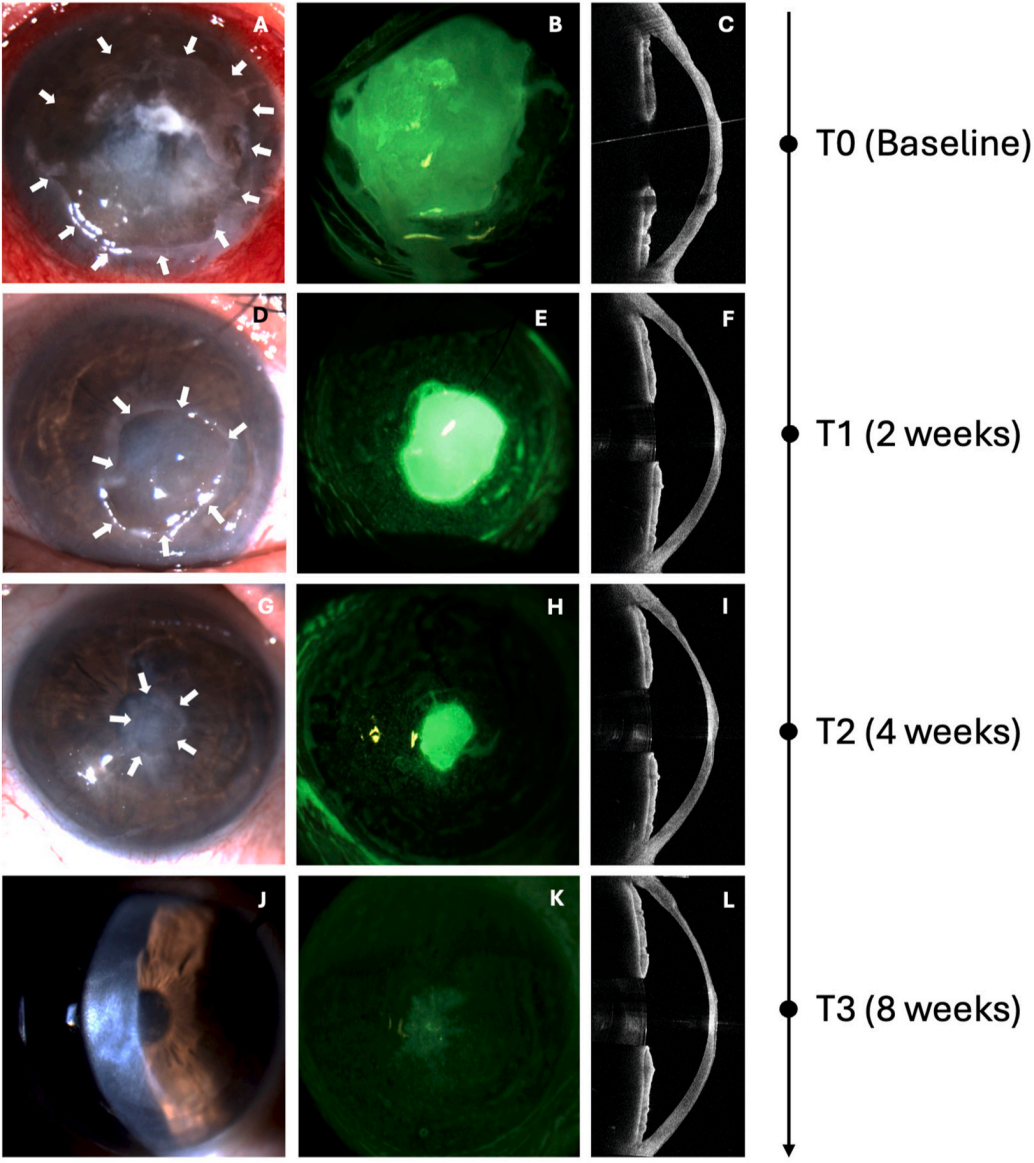
Slit lamp photographs and anterior segment-optical coherence tomography (AS-OCT) scans of the corneal lesion through the follow up period. White arrows delineate the margins of the corneal persistent epithelial defect (PED). A, B and C, slit-lamp photographs of the cornea with and without yellow filter and AS-OCT scan at T0. D, E and F, slit-lamp photographs of the cornea with and without yellow filter and AS-OCT scan at T1. G, H and I, slit-lamp photographs of the cornea with and without yellow filter and AS-OCT scan at T2. J, K and L, slit-lamp photographs of the cornea with and without yellow filter and AS-OCT scan at T3. (For interpretation of the references to colour in this figure legend, the reader is referred to the Web version of this article.)

**Fig. 2:**
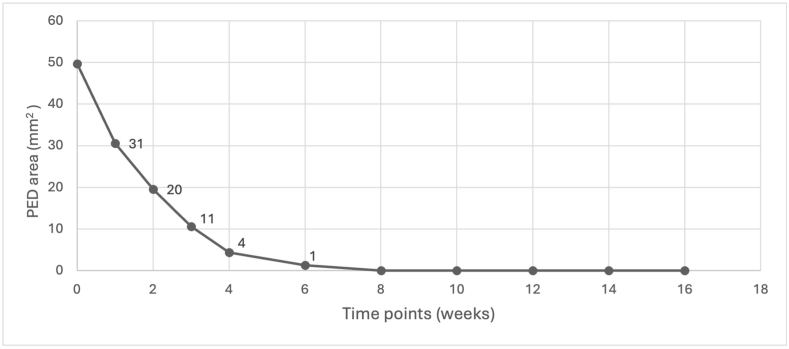
Graph showing the changes of the area of the epithelial defect (expressed in mm^2^) throughout the entire follow-up period. The size of the epithelial defect was digitally analyzed using the public domain software ImageJ 1.53t (National Institutes of Health, Bethesda, MD).

A 52-year-old patient with a history of herpes simplex virus keratitis was referred to our University Eye Clinic for a recalcitrant NK in the right eye. In the previous 4 weeks, multiple topical treatments (antibiotic eye drops, tear substitutes and ointments) have been prescribed along with oral antivirals (acyclovir 800 mg 3 times daily) without any benefit. Apart from previous viral episodes, no history of diabetes or other autoimmune disorders potentially impairing the ocular surface status was reported; eyelid anatomy and function were normal. Upon presentation, BCVA was limited to hand motion. Slit-lamp examination showed marked conjunctival hyperemia (Efron grade IV), a large corneal ulcer with irregular shape, central stromal melting, and 360-degree corneal neovascularization extending from the limbus toward the central cornea (Fig. 1A). Corneal fluorescein staining highlighted the presence of a PED whose area measured 49.7 mm^2^ (width of 7.7 mm and height of 9.0 mm) (Fig. 1B). AS-OCT showed an irregular corneal surface with a paracentral hyperreflective area corresponding to the keratolysis (Fig. 1C). The CTP measured 412 μm. Corneal sensation evaluated by Cochet–Bonnet esthesiometer was completely absent (0 mm) in all quadrants. Topical therapy was discontinued due to lack of efficacy and to avoid potential interference from preservatives or excipients that could delay or hinder corneal healing. Oral antiviral treatment was maintained unchanged and insulin eye drops (1 U/mL) were prescribed 4 times daily. To enhance the retention of the drug over the ocular surface, cross-linked HA canalicular gel was inserted in the lower punctum. The patient was then followed up biweekly. After 2 weeks (T1), conjunctival hyperemia was reduced (Efron grade III), as well as the area of the PED (19.6 mm^2^; width of 5.0 mm and height of 4.9 mm) (Fig. 1D and F). AS-OCT showed a more homogenous corneal surface (Fig. 1E) with a CTP of 295 μm. After 4 weeks (T2), a further reduction of conjunctival hyperemia (Efron grade II) and PED area (4.4 mm^2^; width of 2.5 mm and height of 2.3 mm) was observed (Fig. 1G and H). The CTP was 288 μm (Fig. 1I). After 8 weeks (T3), a complete closure of the PED was reached with slight residual conjunctival hyperemia (Efron grade I) (Fig. 1J and K). AS-OCT confirmed the presence of a regular corneal surface with a stromal hyperreflective signal corresponding to residual scarring (Fig. 1L) and a CTP of 269 μm. The BCVA improved to counting finger at 1 m Fig. 2 shows the changes over time of the area of corneal PED. The patient continued to instill insulin eye drops for 2 additional weeks after complete healing, then switched to unpreserved tear substitutes. Currently, 8 weeks after complete healing, the corneal epithelium continues to be integrum. No ocular or systemic side effects were observed during the entire follow-up period.

## 4 Discussion

In the present case report, the combined use of insulin eye drops and cross-linked HA canalicular gel demonstrated a high efficacy in achieving epithelial healing in a recalcitrant case of severe NK. The progressive reduction of the PED, culminating in complete re-epithelialization within 8 weeks of treatment initiation, further corroborates the therapeutic potential of topical insulin in the context of complex corneal pathology. Moreover, these findings underscore the clinical utility of using a canalicular gel occlusive device as a tool to enhance drug residence time over the ocular surface, thereby potentiating the therapeutic effect.

The absence of well-established and universally accessible therapeutic options makes the medical management of NK particularly challenging.^2^^,^^5^ In this context, topical insulin therapy has recently gained considerable attention as an effective agent for promoting corneal re-epithelialization in patients unresponsive to conventional treatments. From a biological standpoint, the rationale for using insulin on the ocular surface is supported by the constitutive expression of insulin and insulin-like growth factor-1 (IGF-1) receptors on corneal epithelial cells. Once activated, these receptors trigger downstream through the PI3-kinase/Akt and the MAP-kinase (ERK 1/2) pathways, which in turn drive cytoskeletal reorganization, accelerate epithelial cell migration toward the wound edge and support proliferation of limbal and basal epithelial cells.^10^ In vitro evidence has consistently shown that exposure of human corneal epithelial monolayers to insulin enhances closure of experimentally induced wounds. In vivo, insulin signaling has additionally been associated with trophic effects on corneal nerves, which may be particularly relevant in NK where the loss of trigeminal neurotrophic support is the central pathogenic event.^2^^,^^3^^,^^15^ Although only a randomized controlled trial has been conducted in patients with PEDs treated with insulin eye drops,^8^ an increasing number of reports (mainly case reports/case series) suggest that topical insulin may constitute a valuable adjunct in the management of this condition. Clinical studies assessing insulin therapy in PEDs of various etiologies have reported epithelial closure rates ranging from 81% to 100% after variable healing times ranging from 3 to 124 days.^7^^,^^9^^,^^15^, ^16^, ^17^, ^18^, ^19^ Notably, this therapeutic agent offers practical advantages, including widespread availability and low cost, while maintaining a favorable safety profile.^7^ Nonetheless, physiological ocular defense mechanisms, such as blinking, tearing and nasolacrimal drainage, inherently limit drug retention on the ocular surface, potentially reducing its therapeutic efficacy. In this regard, the use of canalicular gel occlusive device represents a rational strategy to prolong the residence time of topical insulin, which may enhance the likelihood and accelerate the rate of re-epithelialization, particularly in eyes with keratolysis at high risk for corneal perforation.

The severity of the case presented herein (Mackie stage III NK) provided a compelling clinical rationale for adopting an aggressive yet conservative combined therapeutic strategy. In such advanced scenarios, the window of opportunity for medical intervention is inherently narrow, as progressive stromal degradation driven by proteolytic enzymes and impaired cellular repair mechanisms may rapidly culminate in structural failure of the corneal wall. Under these circumstances, expediting re-epithelialization is not merely a functional objective but an anatomical imperative, as restoration of an intact epithelial barrier is essential to arrest the enzymatic cascade responsible for stromal dissolution. Topical insulin, through its putative actions on epithelial cell migration and corneal nerve regeneration, directly addresses this requirement. However, in eyes with keratolysis, the already impaired ocular surface architecture, combined with heightened reflex tearing and inflammatory exudation, may further impair drug retention and reduce the bioavailability of topically administered agents. In this context, the adjunctive use of canalicular gel occlusive device appears of particular significance. Indeed, by occluding the canalicular drainage pathway, the cross-linked HA gel prolongs the residence time of the instilled insulin on the ocular surface, thereby increasing both the duration and the local concentration of receptor stimulation. In this perspective, the canalicular gel does not merely act as an inert tear-conserving device, but functions as a pharmacokinetic amplifier of the biological activity of insulin, plausibly translating into a more robust and sustained pro-epithelialization signal in eyes in which rapid drug washout would otherwise blunt its therapeutic potential. This pharmacokinetic advantage may be especially relevant in severe cases in which a prompt and sustained biological response is critical to prevent irreversible structural damage and avoid the need for more invasive surgical interventions.

Unlike conventional punctal plugs, this gel-based delivery system offers a minimally invasive approach to enhance tear film stability, with potential advantages in terms of patient comfort, ease of administration, and diminished risk of device-related complications, such as plug extrusion.^12^^,^^13^ Recent investigations have demonstrated that canalicular gel occlusive device effectively alleviate the symptoms of dry eye and ameliorate key clinical parameters, including tear film stability and ocular surface integrity, showing noninferiority compared to conventional plugs. In addition, patient-reported outcome questionnaire has further substantiated the favorable tolerability profile of canalicular gel occlusive device, with over 80% of participants reporting an absence of pain and minimal adverse events, supporting its role as a viable therapeutic strategy.^12^^,^^13^

Several agents have demonstrated meaningful efficacy in promoting corneal re-epithelialization and are incorporated into the current therapeutic armamentarium of PEDs.^5^ Cenegermin (rhNGF), the only treatment specifically approved for NK, has demonstrated efficacy, achieving complete corneal healing in a substantial proportion of patients with moderate-to-severe disease. However, the limited access in some countries due to high cost and stringent cold-chain storage requirements represents a significant barrier to widespread clinical adoption.^20^ Blood-derived preparations, including autologous serum eye drops and platelet-rich plasma (PRP), represent another well-established category of pro-epithelialization agents, providing a spectrum of growth factors, cytokines, and neurotrophic mediators that mimic the composition of natural tears and support ocular surface repair.^21^ Similarly, amniotic membrane, whether as a sutureless amniotic membrane or as amniotic membrane extract eye drops (AMEED), has been widely employed in PEDs refractory to conventional treatment, exerting anti-inflammatory, anti-scarring, and pro-regenerative effects on the compromised ocular surface.^7^^,^^22^ Although it is conceivable that the combination of any of the aforementioned approaches with canalicular gel occlusive device might similarly confer favorable clinical outcomes through enhanced drug retention, practical accessibility, low cost, and increasing evidence supporting the efficacy of topical insulin represent compelling arguments for its preferential use in this context.

This case report describes the successful management of a severe NK through the combined use of topical insulin and canalicular gel occlusive device. The complete re-epithelialization achieved within 8 weeks, along with the resolution of active stromal melting, highlights the potential clinical benefit of this novel combined approach in cases refractory to conventional therapies. While the relative contribution of each component cannot be definitively determined from a single case observation, the rationale for combination therapy is supported by complementary mechanisms of action. On the one hand, topical insulin acts through its neuro-regenerative and pro-migratory properties; on the other hand, canalicular occlusion allows the prolongation of drug residence time over the diseased ocular surface. Although insulin monotherapy has shown efficacy in PEDs of various etiologies, the features of the case presented herein (stage III corneal ulcer with keratolysis and marked reflex tearing) may have limited its success. In this context, the canalicular gel was introduced not as an alternative therapeutic agent, but rather to sustain an effective concentration of insulin at the wound interface. Whether monotherapy would suffice in a less impaired ocular surface remains to be determined.

This study has several limitations that should be acknowledged. Firstly, the evidence provided derives from a single patient, and the findings therefore cannot be extrapolated to the broader population of subjects with severe NK. Individual factors, including the specific etiology, the duration and nature of previous unsuccessful treatments, and the patient's intrinsic reparative capacity, may all have contributed to the favorable outcome, thereby limiting the external validity of our observations. Secondly, since topical insulin and cross-linked HA canalicular gel were initiated simultaneously, the relative contribution of each component to the final clinical result cannot be determined. Thirdly, although the canalicular gel occlusive device employed in this case offers a minimally invasive and well-tolerated means of prolonging ocular surface drug retention, its cost and accessibility may constitute barriers to widespread adoption in low-income countries.

Overall, these preliminary findings support the potential value of the proposed synergistic strategy, but prospective, controlled studies in larger cohorts are warranted to better define its efficacy, optimal dosing, and long-term safety.

## Claim of priority

After conducting a literature review on March 23, 2026 utilizing PubMed, Google Scholar, and other search engines, using the key words “Canalicular gel, Neurotrophic keratopathy, Topical insulin”, we did not find any prior reports.

## Ethics approval and consent to participate

Formal ethics committee approval was not required for a single patient. This case report was conducted in accordance with the Declaration of Helsinki. Written informed consent was obtained from the patient.

## Consent for publication

Written informed consent was obtained from the patient for publication of this case report and any accompanying images.

## Availability of data and materials

No datasets were generated or analyzed during the current study.

## Artificial intelligence (AI) statement

AI was not used in the preparation of the manuscript.

## Funding

Funded by 10.13039/100013003University of Cagliari under the call “Open Access grants: call for applications for contributions to the open access publication 2026”.

## CRediT authorship contribution statement

**Filippo Lixi:** Conceptualization, Data curation, Formal analysis, Funding acquisition, Investigation, Methodology, Software, Validation, Visualization, Writing – original draft, Writing – review & editing. **Camilla Bianchi:** Conceptualization, Data curation, Formal analysis, Investigation, Validation, Visualization, Writing – original draft, Writing – review & editing. **Claudia Corda:** Conceptualization, Data curation, Formal analysis, Investigation, Writing – original draft, Writing – review & editing. **Mario Verdiglione:** Methodology, Resources, Visualization. **Giulia Coco:** Methodology, Supervision, Validation, Visualization, Writing – original draft, Writing – review & editing. **Giuseppe Giannaccare:** Conceptualization, Funding acquisition, Methodology, Project administration, Resources, Supervision, Validation, Visualization, Writing – original draft, Writing – review & editing.

## Declaration of competing interest

The authors declare that they have no known competing financial interests or personal relationships that could have appeared to influence the work reported in this paper.
